# Seminal cadmium affects human sperm motility through stable binding to the cell membrane

**DOI:** 10.3389/fcell.2023.1134304

**Published:** 2023-05-18

**Authors:** Claudia Pappalardo, Ilaria Cosci, Giulia Moro, Angela Maria Stortini, Annalisa Sandon, Cristina De Angelis, Giacomo Galdiero, Marco Trifuoggi, Rosario Pivonello, Federica Pedrucci, Andrea Di Nisio, Carlo Foresta, Alberto Ferlin, Luca De Toni

**Affiliations:** ^1^ Department of Medicine, Unit of Andrology and Reproductive Medicine, University of Padova, Padova, Italy; ^2^ Veneto Institute of Oncology IOV—IRCCS, Padova, Italy; ^3^ Department of Molecular Sciences and Nanosystems, Ca’ Foscari University of Venezia, Venezia, Italy; ^4^ Department of Civil, Environmental and Architectural Engineering—ICEA—Laboratories, University of Padova, Padova, Italy; ^5^ Dipartimento di Medicina Clinica e Chirurgia, Sezione di Endocrinologia, Unità di Andrologia e Medicina della Riproduzione e della Sessualità Maschile e Femminile (FERTISEXCARES), Università Federico II di Napoli, Naples, Italy; ^6^ Dipartimento di Scienze Chimiche, Università Federico II di Napoli, Naples, Italy; ^7^ Staff of Unesco Chair for Health Education and Sustainable Development, Federico II University, Naples, Italy

**Keywords:** heavy metals, ionic mimicry, scanning electron microscopy/energy dispersive spectroscopy, scavenging, inductively-coupled plasma spectroscopy

## Abstract

Environmental pollutants are claimed to be major factors involved in the progressive decline of the fertility rate worldwide. Exposure to the heavy metal Cadmium (Cd) has been associated with reproductive toxicity due to its ionic mimicry. However, the possible direct accumulation of Cd in human sperm cells has been poorly investigated. In this study, we aimed to clarify the possible direct effect of Cd exposure on sperm function through the analysis of its cell accumulation. Semen samples from 30 male subjects residing in high environmental impact areas and adhering to the “Exposoma e Plurifocalità nella Prevenzione Oncologica” campaign for testis cancer prevention were compared with semen samples from 15 males residing in low exposure areas. Semen levels and cell Cd content were quantified by inductively coupled plasma (ICP) spectroscopy. Cell Cd distribution was assessed by scanning electron microscopy coupled with energy dispersive spectroscopy (SEM-EDS). The impact of Cd on sperm function was evaluated by the *in vitro* exposure to the heavy metal, whilst possible scavenging approaches/agents were assessed. In addition to higher values of semen Cd, exposed subjects showed a reduction in total motile sperm fraction compared to not-exposed controls (59.6% ± 13.6% vs*.* 66.3% ± 7.3%, *p* = 0.037). Semen Cd levels were also significantly correlated with SEM-EDS signals of Cd detected on the head and neck of sperm (respectively *p* = 0.738, *p* < 0.001 and *ρ* = 0.465, *p* < 0.001). A total of 2 h of *in vitro* exposure to 0.5 μM Cd was associated with a significant reduction of sperm progressive motility. Scavenging approaches with either hypo-osmotic swelling or 10 μM reduced glutathione were ineffective in blunting cell Cd and restoring motility. The reduction of exposure levels appears to be the main approach to reducing the reproductive issues associated with Cd.

## Introduction

Environmental factors are recognized as major determinants of disease from metabolic disorders to cardiovascular diseases and cancer (Prüss-Üstün et al., 2016). Similarly, environmental factors are claimed to explain the progressive global decline in the fertility rate observed over the past 5 decades (Skakkebæk et al., 2022). Particularly in humans, men appear more susceptible than women to the disruption of fertility due to environmental exposure to several pollutants associated, for example, with specific lifestyles or occupations (Skakkebæk et al., 2022). Among the environmental pollutants recognized to have a detrimental role in male fertility, heavy metals, particularly cadmium (Cd), occupy a relevant position (Kumar et al., 2014; Stortini et al., 2020). Cadmium is a relatively rare heavy metal found almost ubiquitously in the soil, whose absorption by plant organisms allows this element to enter the food chain, thus contributing to human exposure (Cao C et al., 2022). In addition, there are recognized conditions associated with an increased risk of exposure to Cd, such as occupation in cadmium extraction/processing plants and smoking habits (Skakkebæk et al., 2022). To this regard, *Nicotiana tabacum* was found to efficiently accumulate bivalent heavy metals, like Cd, in its leaves, and the subsequent production process of dried tobacco leaves-based products generate, in fact, concentrated sources of these potentially toxic agents by inhalation of combustion aerosols (Cao C et al., 2022). Another recognized source of exposure to Cd is the consumption of vegetables and soil products grown in areas heavily polluted by this metal (Esposito et al., 2018). The reproductive toxicity associated with Cadmium exposure is mediated by multiple mechanisms, largely uncovered in experimental *in vitro* and *in vivo* animal models, including structural damage to testis vasculature and the blood–testis barrier, oxidative stress and inflammation, Sertoli and Leydig cells-directed cytotoxicity, epigenetic regulation of genes involved in male reproductive function, endocrine disruption of the hypothalamus–pituitary–testis axis, and interference with essential ions (de Angelis et al., 2017).

With reference to the latter mechanism, the toxicity of Cd is strictly related to its electronic characteristics, in terms of bond diameter and valence, which make it similar to calcium (Ca) and zinc (Zn), a phenomenon known as ionic mimicry (Thévenod et al., 2019). In addition, Cd is extremely affine to sulfur, enabling a thorough interaction with sulfide, thiol groups, and sulfur-rich complex compounds and resulting in the disruption of the cellular processes in which these compounds participate (Helbig et al., 2008). On the other hand, Cd is highly persistent in humans where a two-compartments model of distribution, applied to plant workers after exposure cessation, evidenced a fast component and slow compartment with estimated serum half-lives of respectively 75–128 days and 7.4–16 years (Jarup et al., 1983). Because of its similarity to Zn, Cd is believed to access specific cell populations and tissues by exploiting Zn-devoted solute carriers, such as ZIP8 and ZIP14, particularly represented in the male genital tract (Foresta et al., 2014; Thévenod et al., 2019). Accordingly, rodent models exposed to Cd via drinking water showed increased metal accumulation in the liver, testes, epididymis and prostatic lobes (Lacorte et al., 2013). In humans, available data showed that Cd is detectable in semen at levels strictly correlated with smoking habits and that seminal Cd levels are inversely related with sperm count, motility, and viability (Bazid et al., 2022). The toxicity of Cd on sperm cells has been typically attributed to the interference with the germline differentiation phase within the testis, through the disruption of tight junctions between Sertoli cells and the imbalance of the cell redox system by altering trace elements homeostasis of the likes of copper (Cu) and zinc (Siu et al., 2009a; Siu et al., 2009b; Nzengue et al., 2011; Lacorte et al., 2013). Additional mechanistic impairment of the cell redox system by Cd has been claimed through the direct disruption of the mitochondrial respiratory chain or through the downstream irreversible oxidative modifications of intracellular antioxidant proteins, such as thioredoxin 1 and peroxiredoxin 1, due to the excessive generation of reactive oxygen intermediates (Cao W et al., 2022; Wang et al., 2022). However, Cd found in seminal plasma, which is essentially prostate-derived, has been suggested to exert possible direct effects even on fully differentiated and functionally mature spermatozoa (Zhao et al., 2017; Ranganathan et al., 2018). In this frame, the current study aims to clarify the possible direct effect of Cd exposure on sperm function through the analysis of cell accumulation of the metal.

## Methods

### Chemicals

Cadmium chloride (Cat# 655198), glutaraldehyde solution (cat# 340855), formaldehyde solution (cat# F8775), and sodium cacodylate trihydrate (cat# C4945) were all purchased from Merk Life Science S.r.l. (Milan, Italy). L-Glutathione in the reduced thiol form (Cat# 6832.3) was purchased from Carl Roth GmbH (Karlsruhe, Germany). Sperm Washing Medium (SWM) was purchased from Fujifilm Italia S.p.A. (Rome, Italy).

### Participant recruitment

Participant recruitment was performed in line with the principles of the Declaration of Helsinki upon approval by the Ethical Committee of Università Federico II di Napoli (Prot. N°158/19). The cohort of the current study included Caucasian males of reproductive age residing in the Campania Region, recruited from 2017 to 2019 within the “Exposoma e Plurifocalità nella Prevenzione Oncologica” research project and funded by Campania Region—POR-FESR 2014-2020. This was an awareness campaign for testis cancer prevention in high environmental impact (HI) areas, which are areas identified on the basis of the Campania Region Environmental Protection Agency (ARPAC) reports as having the highest concentration of illegal disposal sites of toxic waste and being characterized by frequent uncontrolled waste incineration. The awareness campaign was promoted by Unità di Andrologia e Medicina della Riproduzione e della Sessualità Maschile e Femminile (FERTISEXCARES)—Dipartimento di Medicina Clinica e Chirurgia, Sezione di Endocrinologia, Università Federico II di Napoli, Naples, Italy (ARPAC, n.d.) by the publication of the initiative on the official website of the FERTISEXCARES center, by social media networks, and by locally distributed flyers. Participants were also consecutively enrolled by actively contacting them by phone, based on telephone directories, and with the support of general practitioners and local pharmacists.

The inclusion criteria for enrolment were as follows: a residence in municipalities belonging to HI areas of the Campania Region and no “*a priori*” selection based on the presence or absence of infertility and/or andrological disorders. Exclusion criteria were as follows: 1) psychological and/or psychiatric disorders; 2) endocrine and/or other systemic diseases; 3) reported history of or concurrent non-therapeutic use of psychotropic substances and/or recreational drugs; 4) reported history of or concurrent alcoholism or suspicion of alcohol abuse. Written informed consent for participation in the study was obtained upon enrolment of participants attending the FERTISEXCARES center. Upon enrolment, a progressive code number was assigned to each participant by the enrolling andrologist. The examining biologist performed semen analysis blinded to participant identity, residential municipality, and clinical characteristics.

In total, 30 males aged 17–44 years were considered for the current study.

Furthermore, an additional 15 normozoospermic subjects attending the Unit of Andrology and Reproductive Medicine, University Hospital of Padova (Padova Italy; protocol number 2208P and successive amendments approved by the Institutional Ethics Committee of the University Hospital of Padova, Italy), residing in low-exposure areas, were additionally enrolled as healthy control donors for the *in vitro* analysis of Cd exposure on seminal plasma free-sperm cells, obtained by centrifugation and washing. Quantitative determination of Cd was performed in whole semen by inductively coupled plasma-mass spectrometry (ICP-MS) (Aurora M90; Bruker) as previously described (Nunzio et al., 2022). Serum levels of total testosterone (T), luteinizing hormone (LH), and follicle-stimulating hormone (FSH) were evaluated by commercial electrochemiluminescence immunoassay methods as previously described (Selice et al., 2011).

### Semen analysis

Semen samples were collected on-site at the FERTISEXCARES center by masturbation directly into a sterile plastic container after 3–5 days of sexual abstinence. Use of medications (antibiotics, antivirals, anabolic hormones, exposure to X rays, etc.) or pathological conditions (fever) with known effects on seminal parameters were accounted for at the time of semen collection by anamnestic interview. Semen samples were analyzed according to the 2010 World Health Organization laboratory manual guidelines (World Health Organization., 2010). After collection, ejaculates were left to liquefy for 30’ at 37°C to obtain complete liquefaction before processing and maintained at 37°C during the analysis. The following seminal parameters were considered for analysis: pH, semen volume (mL), sperm concentration (10^6^ cells/mL), total count (10^6^ cells/ejaculate), total motility (%), progressive motility (%), *in situ* motility (%), immotile spermatozoa (%), typical sperm morphology (%), atypical sperm morphology (%), and sperm viability (%). Sperm count and motility were evaluated with the Makler Counting Chamber (Sefi Medical Instruments, Haifa, Israel) using a Nikon E200 optical microscope (Nikon, Tokyo, Japan) (×20 magnification, phase-contrast); sperm morphology was evaluated after Giemsa staining (×100 magnification, bright field); sperm viability was evaluated after Eosin-nigrosine staining (×100 magnification, bright field). World Health Organization, (2010) criteria for normozoospermia were as follow: sperm total count ≥39×10^6^/ejaculate or sperm concentration ≥15×10^6^/mL; progressive sperm motility ≥32% or total sperm motility ≥40%; normal sperm morphology ≥4% typical forms; sperm viability ≥58% viable cells. All semen samples were analyzed in the same laboratory by the same trained and experienced operator. The laboratory and all the operators take part in an international external quality control program for semen analysis, provided by United Kingdom Neqas International Quality Expertise, on a quarterly basis. Aliquots of 500 µL of whole semen were stored in metal-free tubes at −80°C for subsequent Cd quantitative determination. The hypo-osmotic swelling procedure was performed as previously described (World Health Organization., 2010).

### Inductively coupled plasma (ICP) spectroscopy

Samples were prepared by a two-step digestion process at room temperature. In a typical procedure, a single pellet of sperm cells was completely dissolved in 1 mL of concentrated Nitric Acid (HNO_3_, RPE reagent 65%, Carlo Erba Reagents S.r.l. Cornaredo, Milan, Italy) and the reaction was allowed to proceed overnight. Thereafter, the solution obtained was treated with 1 mL of hydrogen peroxide (H_2_O_2_, Baker Analyzed Reagent 30%, Carlo Erba Reagents S.r.l.), and deionized water was added up to a final volume of 5 mL.

Cd concentration measurements were carried out by an Inductively Coupled Plasma Optical Emission Spectroscopy analyzer (ICP-OES Optima 4200 DV, PerkinElmer). The ICP-OES principle is based on the fact that atoms and ions can absorb energy from argon plasma (10,000 K) to move electrons from the ground state to an excited one. The excited atoms release electromagnetic radiations at a specific wavelength as they return to a lower energy level. Mixtures of different atoms in the sample can be distinguished on the base of the atom-specific wavelength emitted and the emitted radiation intensity is proportional to the number of atoms or ions undergoing the energy transition. The Beer–Lambert law describes the relationship between light intensity and concentration of the element.

### Cd distribution mapping on sperm cells

Scanning electron microscopy (SEM) coupled with energy dispersive spectroscopy (EDS) were applied to map Cd distribution in sperm cells in order to characterize the possible site of Cd accumulation. SEM-EDS analysis were performed using a TM3000 Hitachi tabletop scanning electron microscope coupled with an X-ray microanalysis system SwiftED3000 (Oxford Instruments Italia, San Giuliano Milanese, Milan, Italy). The EDS spectra were acquired with an acquisition time of 30 s, a process time of 5 s and an accelerating voltage of 15 kV. SEM topological images were acquired using both back scattered electrons (BSE) and secondary electrons (SE), as reported in SEM images captions, in order to study sperm cells morphology prior to map Cd distribution. The estimated scanning depth, according to the applied conditions, was estimated as closer to 1 µm. Spermatozoa obtained from healthy normozoospermic subjects having semen content of Cd < LOD were studied to validate the protocol. In particular, basal cells were used as not-exposed controls whilst cells exposed *in vitro* to Cd 0.5 μM for 1 hour at 37°C, followed by thorough washing with PBS, were used as Cd exposed samples. Prior to proceed with the analysis, all sperm cells were isolated by centrifugation, washed in phosphate buffer solution (PBS) and fixed with 2.5% glutaraldehyde solution in cacodylate buffer. The cell suspension was then drop-casted on filter paper, air dried and then coated with sputtered gold. All samples underwent gold coating, obtained by a Cressington 108 auto sputter coater (Watford, United Kingdom) with a target distance of 50 mm, applying a current of 40 mA for 30 s under Argon atmosphere. The element ratios were calculated with a standardless approach using the SwiftED3000 software (Oxford Instruments), and the results obtained were qualitative. The Cd content was estimated after normalizing its weight percentage (wt%) with the one of carbon considered as a housekeeping value. The different regions of the sperm cells, namely, the head, neck, and tail, were identified based on the cell morphology. These three area(s) of interest (AOI) were discriminated as follows: the sperm head region was considered as included among the borders between the sperm cells and the media and between the post-acrosomal sheath and the upper limit of the neck. The neck AOI ranged from the posterior ring to the lower end of the mitochondria sheath and between lateral borders corresponding to the cell/media interface. The tail AOI was considered as the principal piece of the flagellum with the lateral borders corresponding to the sperm cells and the media interface. In turn, in all AOI, Cd levels were estimated via energy dispersive spectroscopy techniques (EDS). For topological evaluation of Cd detection on sperm cells by SEM-EDS, field selection bias within samples was avoided by adopting a non-random, non-human-chosen, image analysis approach; specifically, the first field selection was operated in a fixed centered position of the sample, thereafter, fields were analyzed at regular intervals within the right and left sides across the sample, by direct evaluation of cells in a defined area by adopting the margins of the sample as guard zones, given that loss of cells, irregular spread or shrinkage of cells, or artifacts, can occur. This approach increases the likelihood that analysis is more representative of the sample as a whole and that sperm cells have an equal probability of being evaluated, irrespective of their status. The mapping of Cd levels was then reported ex post as color scale dots according to the results of the analysis.

### Statistical analysis

Statistical analyses were performed using SPSS 23 Statistics for Windows (Chicago, IL). The results were expressed as means ± standard deviation (SD). The Shapiro-Wilk test was used to verify normal distribution of continuous variables. The comparison of the mean values of two groups was performed by Student’s t-test. Analyses of differences between the mean value of more than two groups were conducted using ANOVA tests with Bonferroni’s correction *post hoc* tests for pairwise comparisons between subgroups. A non-parametric Mann-Whitney *U* test was used to compare two groups of continuous variables with non-normal distribution. The correlation of continuous variables was assessed using Person’s correlation test. Spearman’s correlation test was used for variables with not-normal distribution. In the case of PAEs semen levels being lower than LOD, LOD/√2 substitutions were adopted for statistical analysis. Values of *p* < 0.05 were considered significant.

## Results

### Subjects characteristics and correlation with semen Cd levels

Clinical and demographic data of the 30 exposed subjects residing in areas with high environmental heavy metals pollution and the 15 age-matched not-exposed control subjects, are reported in Table 1. The comparison of the age, BMI, and semen parameters between the two groups showed no significant differences, except for the percentage of motile sperm cells, showing higher values in not exposed compared to exposed subjects (respectively 66.3% ± 7.3% vs. 59.6% ± 13.6%, *p* = 0.037). As expected, exposed subjects had significantly higher semen Cd levels compared to not-exposed controls (respectively: < LOD not exposed, 1.4 ± 0.9 μg/L exposed, *p* = 0.001 at both parametric Student’s t-test and non-parametric Mann-Whitney *U*-test). The evaluation of the possible correlation between semen Cd levels and sperm parameters showed no significant evidence when the analysis was applied to all study participants or restricted to the group of exposed subjects only (all *p* values >0.05, Supplementary Table S1).

**TABLE 1:** Demographic and semen parameters of 30 subjects residing in high environmental impact, at high risk of exposure to heavy metals, and 15 control subjects residing in low exposure areas.

Parameter	Not exposed (*n* = 15) mean value ±SD (Median, Interquartile range)	Exposed (*n* = 30) mean value ±SD (Median, Interquartile range)	*p*-value
Age (years)	35.1 ± 6.3 (34.0, 6.1)	31.2 ± 7.9 (30.0, 8.3)	0.223
			0.251
BMI (kg/m^2^)	27.3 ± 4.9 (27.1, 5.0)	26.7 ± 4.5 (27.0, 5.3)	0.684 [0.711]
Semen volume (mL)	3.9 ± 1.8 (3.5, 1.4)	4.5 ± 1.7 (4.45, 2.25)	0.279
			0.220
Semen pH	8.5 ± 0.4 (8.6, 0.4)	8.4 ± 0.3 (8.4, 0.4)	0.621
			0.325
Total Sperm count (10^6^ cells/ejaculate)	138.4 ± 26.7 (103.0, 46.8)	118.6 ± 105.6 (87.0, 137.93)	0.099
			0.086
Progressive motile sperm fraction (%)	59.1 ± 9.6 (56.0, 20.0)	52.7 ± 14.5 (56.0, 24.5)	0.130
			0.904
Total motile sperm fraction (%)	66.3 ± 7.3 (65.0, 6)	59.6 ± 13.6 (55.0, 18.5)	**0.037**
			**0.048**
Sperms with normal morphology (%)	5.8 ± 2.3 (4.8, 4.0)	7.2 ± 3.3 (7.0, 6.0)	0.101
			0.217
Viable sperm cells (%)	73.0 ± 12.1 (71.0, 17)	71.7 ± 7.6 (72.1, 9.0)	0.765
			0.945
Semen Cd (μg/L)	< LOD (n.a.)	1.4 ± 0.9 (7.0, 1.08)	**0.001**
			0.001*

Abbreviations: standard deviation, SD; body mass index, BMI; Cadmium (Cd). Significant *p* values are in bold. *p* values from non-parametric Mann-Whitney U tests are in *Italics*.

### Characterization of Cd accumulation in human spermatozoa

**FIGURE 1:**
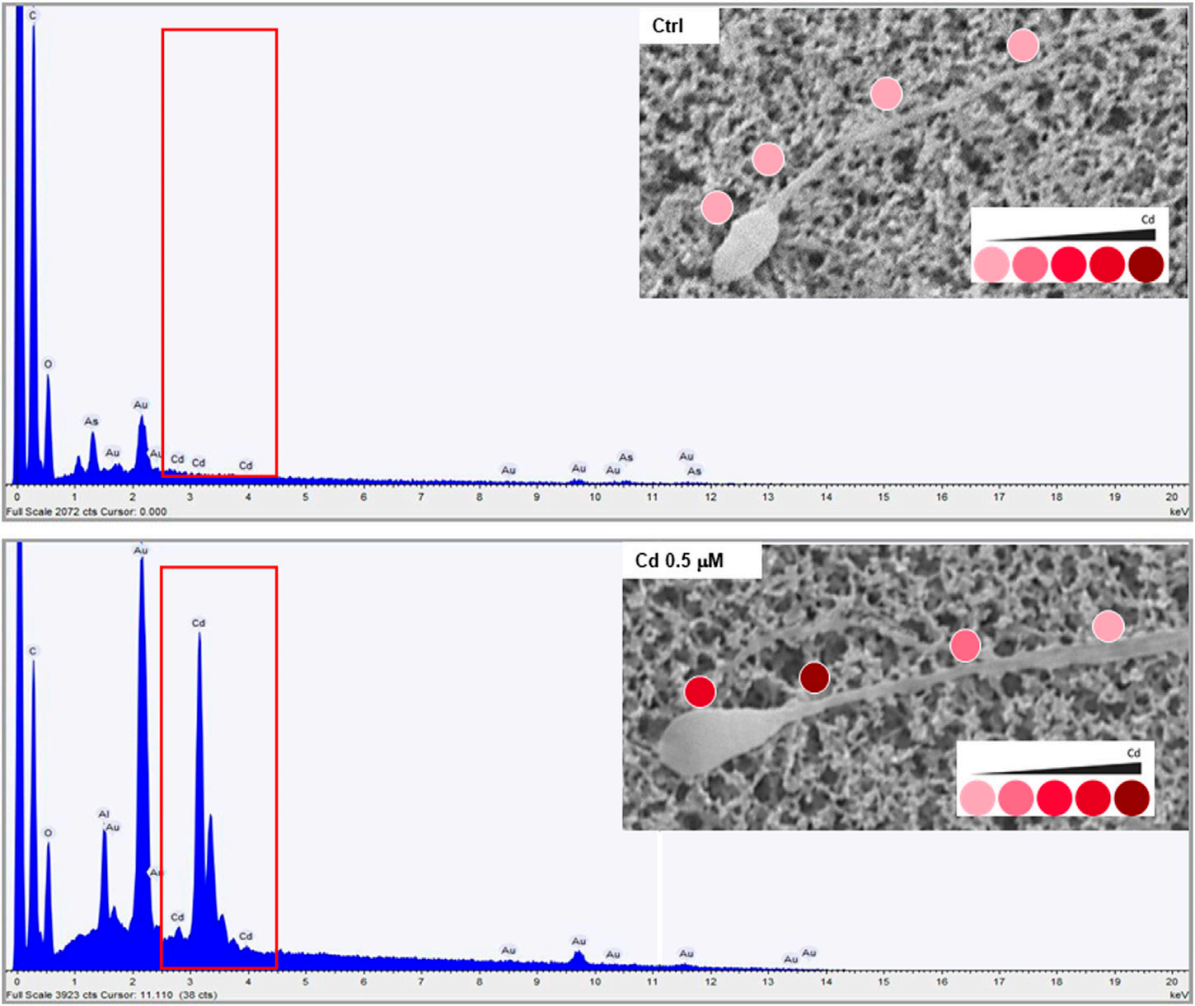
Representative images of Cadmium (Cd) mapping at scanning electron microscopy coupled with energy dispersive spectroscopy (SEM-EDS) analysis in sperm cells from normozoospermic healthy donors at basal not exposed conditions (CTRL) and upon *in vitro* exposure to Cd 0.5 μM for 1 hour at 37°C. The red boxes in the spectra underline the specific signals deriving from Cd detection in elemental analysis by SEM-EDS. The images in the inserts show the levels of Cd detection on the cell through a color intensity scale.

**FIGURE 2:**
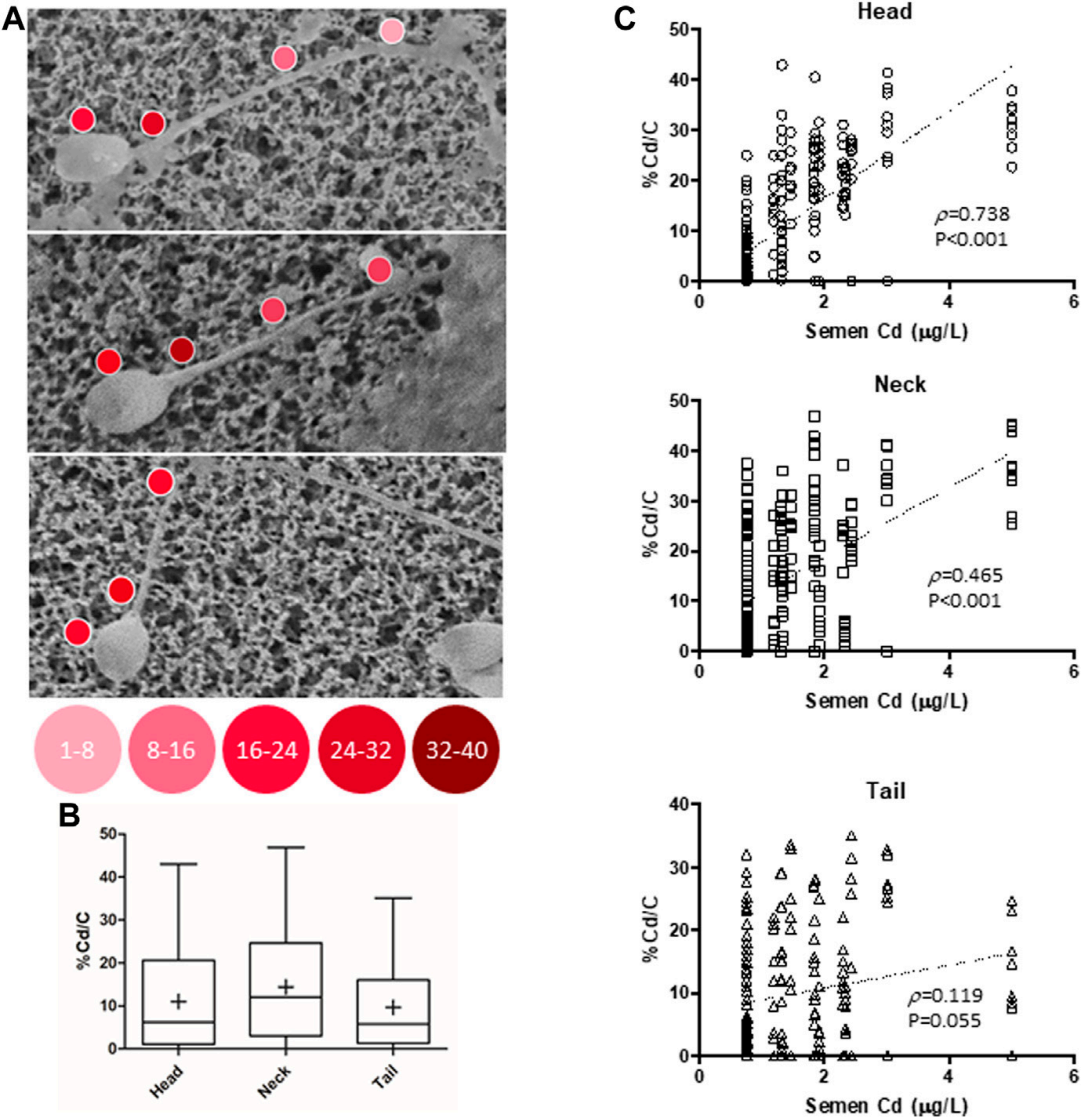
**(A)** Representative images of Cadmium (Cd) mapping at scanning electron microscopy coupled with energy dispersive spectroscopy (SEM-EDS) analysis, conducted on isolated sperm cells from 30 male subjects residing in high environmental impact areas. **(B)** Analysis of Cd signal reported as the percentage of carbon (C) signals in 747 representative cell sites, further distinguished into head, tail, and neck cell domains (249 mearurement each). **(C)** Correlation analysis of the Cd/C% signals, with semen Cd levels, for each cell domain. *p* values refer to Spearman’s correlation test.

In order to characterize the possible accumulation of Cd on sperm cells, a joint approach of cellular content quantification and topological mapping of Cd was performed with, respectively, ICP spectroscopy and SEM-EDS (Figure 1). Sperm cells were isolated from normozoospermic subjects, as negative controls for semen Cd detection, and exposed to Cd 0.5 μM in SWM for 1 h at 37°C. ICP analysis showed that *in vitro* Cd exposure was associated with significant increase of sperm Cd content even after thorough cell washing procedure (respectively < LOD not exposed controls vs*.* 118.6 ± 18.6 ng Cd/10^6^ cells to Cd 0.5 μM, *p* = 0.0247). The corresponding topological evaluation of Cd detection on sperm cells by SEM-EDS retrieved specific Cd signals, supported by EDS-dependent elemental analysis, on cells exposed to Cd 0.5 μM (Figure 1). A further analysis of Cd distribution was applied with the aim to address any site-specific and concentration-dependent Cd accumulation on sperm cells (Figure 2). In order to provide a normalized evaluation of Cd detection, the Cd signal was reported as the percentage of the carbon (C) signal, which has been shown to be relatively stable and consistent across all samples and was therefore considered as a “housekeeping” signal. The punctual evaluation of Cd detection on 747 representative cell sites on isolated sperm cells from environmentally exposed subjects showed highly variable levels of the metal distribution along the cell topology (Figure 2A). The gross distinction of sub-cellular domains into head, neck, and tail (*n* = 249 punctual measurement at each domain) showed higher levels of Cd signal on the sperm neck (respectively: head 11.6 ± 11.4% Cd/C, neck 14.8 ± 12.6% Cd/C, tail 9.8% ± 9.7% Cd/C, *p* < 0.001 at Kruskal-Wallis test for independent samples, Figure 2B). In addition, the signals for Cd on the sperm head and neck, but not tail, were significantly correlated with semen Cd levels (respectively: head *p* = 0.738, *p* < 0.001; neck *p* = 0.465, *p* < 0.001; tail *p* = 0.119, *p* = 0.055; Figure 2C).

**FIGURE 3:**
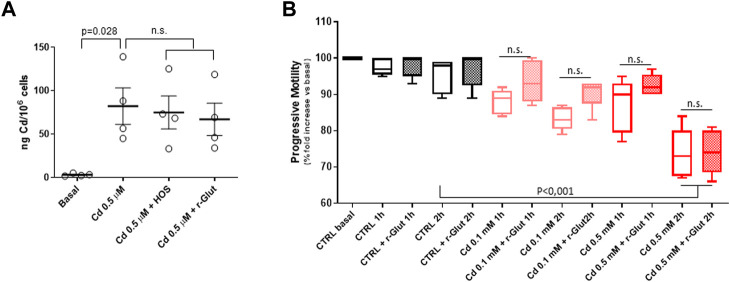
**(A)** Induction coupled plasma spectroscopy analysis of cell Cadmium (Cd) content in sperm cells from normozoospermic healthy donors (*n* = 4) at basal (CTRL), after *in vitro* exposure to Cd 0.5 μM in for 1 hour at 37°C, and after further scavenging treatment with hypo-osmotic swelling (HOS) procedure or after 1-h treatment with 10 μM reduced glutathione (r-Glut). **(B)** Effect of Cd exposure (1 h at 37°C at 0.1 or 0.5 μM) on sperm progressive motility, monitored up to 2 h after exposure. In control condition (CTRL), Cd was omitted. Data are reported as percentage fold increase in the common basal condition. The effect of 10 μM r-Glut was also evaluated. Each experiment was performed on five different normozoospermic healthy donors. Significance: *p* values among the indicated condition are reported; n.s. = *p* > 0.05 between the indicated conditions.

In order to better characterize the affinity for Cd with the sperm cell binding sites, two scavenging approaches were applied: the hypo-osmotic swelling (HOS) procedure and the treatment with reduced glutathione (r-Glut). Accordingly, sperm cells isolated from normozoospermic subjects, and negative for semen Cd detection, were exposed to Cd 0.5 μM in SWM for 1 h. Thereafter, cells underwent HOS or treatment with 10 μM r-Glut for 2 h, and the cell content of Cd was evaluated by ICP (Figure 3A). As expected, exposure to Cd 0.5 μM was associated with significant increase of the metal content in sperm cells (basal 3.1 ± 1.5 ng/10^6^ cells, Cd 0.5 μM 82.2 ± 42,1 ng Cd/10^6^ cells, *p* = 0.028). However, neither HOS nor r-Glut treatment could significantly reduce the Cd load on sperm cells (respectively: Cd 0.5 μM + HOS 74.9 ± 37.9 ng Cd/10^6^ cells, Cd 0.5 μM + r-Glut 67.0 ± 37.4 ng Cd/10^6^ cells; *p* = 0.990 and *p* = 0.919 vs*.* Cd 0.5 μM; Figure 2A).

### Effect of Cd exposure on sperm motility

The impact of Cd exposure on sperm motile function and the possible effect of the scavenging procedure was evaluated. To this regard, since the application of HOS itself, although not affecting cell viability, severely impairs sperm motility, only the treatment with r-Glut 10 μM was considered (Check and Check, 1991). Sperm cells were then exposed for 1 h to Cd 0.1 μM or 0.5 μM, followed or not by 1-h treatment with r-Glut, and the percentage of cells with progressive motility was monitored for a further 2 h. Control samples followed the same treatment with the exception of Cd exposure (Figure 3B). Despite a trend towards a time and concentration-dependent reduction of sperm progressive motility upon Cd exposure, the statistical significance, compared to the not-exposed control condition, was observed only after 2 hours of exposure to Cd 0.5 μM. However, in all three conditions, r-Glut 10 μM was essentially ineffective in resuming sperm motility (all *p* values > 0.05 vs*.* 10 μM r-Glut treatment).

## Discussion

The results of the current study provide evidence that the *in vitro* exposure to Cadmium is associated with the reduction of sperm motility, involving the interaction between the heavy metal and the cell membrane. Importantly, treatment with a sulphur-donor decoy reagent with possible Cd scavenging properties, such as reduced glutathione, is not associated with neither a reduction of the cell content of Cd nor an improvement of cell motility, suggesting some stable interaction with the plasma membrane.

The environmental influence on human health is currently one of the most pursued avenues of investigation of recent years (Soga and Gaston, 2021). In this context, among the recognized environmental pollutants, Cd is a heavy metal widespread in nature whose toxicity justifies careful regulation of exposure by International Health Agencies (EFSA, 2009). Toxico-dynamic of Cd, namely, the molecular mechanisms related to the metal toxicity, largely relies on its high affine to sulfur (Helbig et al., 2008) whilst its toxico-kinetic properties, or the mechanisms by which it distributes and accumulates throughout the body, are related to the ionic mimicry with Ca and Zn ions (Thévenod et al., 2019). The similarity to Zn is probably a key point in Cd toxicity. In fact, Zn is a trace element with an extremely important role for the development and function of the male reproductive system and it is no coincidence that, at testis and accessory sexual glands levels, there is a redundancy of expression of transport proteins involved in the trafficking of Zn (Foresta et al., 2014). Whilst ZnT-type Zn transporters generally exhibit a clear selectivity for Zn ions over Cd, ZIP type Zn transporters ZIP8 and ZIP14 are permeable to other metals, including Cd, whose transport is dependent on extracellular bicarbonate (Hoch et al., 2012; Thévenod et al., 2019). Accordingly, in addition to the liver, mouse models exposed to dietary Cd accumulate the metal in the testis, epididymis, and prostate (Lacorte et al., 2013). The actual competition between Zn and Cd on shared transport systems is supported by numerous experimental data in animal models, according to which Zn supplementation would alleviate cadmium toxicity (reviewed in Peraza et al., 1998).

In the current study voluntary participants residing in areas exposed to heavy metal pollution displayed significantly higher levels of semen Cd compared to not exposed subjects. Of note, exposed subjects had essentially a significant reduction of total motile sperm fraction compared to the unexposed control population. In agreement with our findings, the impairment of sperm parameters, and particularly cell motility, has been observed in patients exposed to environmental Cd by Bazid et al. (Bazid et al., 2022). Evaluating semen parameters in a group of male heavy smokers (classified as smoking more than 20 cigarettes per day), authors documented a significant reduction of sperm motility, viability, and normally formed sperm compared to age-matched non-smokers. Importantly, all three parameters were inversely related to semen Cd levels rather than to serum Cd levels. Most importantly, in the present study semen levels of Cd in exposed subjects were lower, indeed nearly halved, compared to the study from Bazid et al. (Bazid et al., 2022) Nonetheless, we showed a significant impairment of sperm motility. This evidence suggests that motility function is probably among the most sensitive parameter to exposure to the heavy metal.

Moreover, the accumulation of Cd in sperm cells has been previously reported by Calogero et al. (Calogero et al., 2021). Evaluating semen parameters in subjects with different heavy metal exposure according to the residence in rural or industrial areas, authors showed a significant reduction of total sperm count and motility compared to the reference population. Importantly, authors showed that Cd was detectable in isolated and washed sperm cells and that sperm Cd accumulation was increased in those subjects with at least one altered sperm parameter. Accordingly, by the use of SEM-EDS technique, we provided a topological characterization of Cd accumulation, compared to data from Calogero et al., showing that Cd is detected on the cell membrane of washed spermatozoa from patients exposed to environmental Cd. This result also confirms and extends very early data from Ranganathan et al. that provided a correlation between human exposure to Cd, Cd accumulation in sperm cells, and alteration of sperm morphology through the impairment of the cell redox system (Ranganathan et al., 2018). In addition, through the topological mapping of the Cd signal, we showed that Cd is likely found on the sperm surface and that the extent of Cd accumulation on the sperm head and neck significantly correlates with semen Cd levels. These data are consistent with previous evidence showing the previous expression, on the same sites, of membrane proteins able to bind divalent metal cations, such as the proton channel HV-1 (HV-1) (Lishko et al., 2010). HV-1 is involved in the proton extrusion from sperm cytoplasm and, in turn, the resulting intracellular alkalinization activates the cation channels of sperm (CatSper) that regulate cell motility (Lin et al., 2021). Importantly Zn, which is highly represented in seminal plasma, is a physiological blocker of HV-1 and the reduction of concentrations in the female genital tract is pivotal for the motility gain during the fertilization process (Lishko et al., 2010). It is thus possible to tentatively speculate that Cd might vicariate Zn in HV-1 inhibition with more stable binding. In support of this hypothesis, the findings of the current study demonstrated that the *in vitro* exposure to Cd is associated with the impairment of sperm motility, confirming previous data from Zhao et al. (Zhao et al., 2017). However, this effect is not reverted by known sulfur-donor scavengers such as r-Glut. Cd is recognized to be poorly bound to free amino acids, whilst more favorable and stable Cd-binding sites are found in proteins because of their cysteine residues (Andersen, 1984). Accordingly, appreciable Cd removal from complexes with metallothionein is observed upon protein breakdown in the kidney (Squibb et al., 1982). In particular, the number of thiol groups in a polypeptide/protein chain has been shown to increase the corresponding affinity constant for the complexes with Cd (Jacquart et al., 2017). This evidence is consistent with our results, showing that the single-cysteine donor r-Glut is not capable of exerting a scavenging effect on spermatozoa.

In addition to the impact on sperm motility, the detection of Cd in semen and that bound to sperm membranes gains particular interest in the light of the recent study by Zhao et al., showing that the rate of embryo pronuclei formation, upon *in vitro* fertilization applied to mouse gametes, was significantly reduced in a dose- and time-dependent manner when spermatozoa were exposed to Cd compared the not-exposed control group (Zhao et al., 2017). Consistently, available epidemiological studies showed that semen Cd levels are independent predictors of negative pregnancy outcomes in *in vitro* fertility procedures (Wu et al., 2008).

We acknowledge some major study limitations. First of all, we did not evaluate the possible intracellular accumulation of Cd. On this basis, the inefficacy of r-Glut on reverting Cd toxicity may rely on the sperm impermeability to r-Glut itself. Cellular trafficking of reduced and/or oxidized r-Glut has been addressed in several cell types through known and putative r-Glut membrane transporters (Reddy et al., 1973; lantomasi et al., 1997; Chen et al., 2000; Fan et al., 2017; Oestreicher and Morgan, 2019). However, the cell uptake of r-Glut by human spermatozoa has not been clarified so far. Since Cd was detected to be likely on the cell surface by SEM-EDS assay, we considered the extracellular surface of sperm membrane as the major SH-donor scavenger target site of r-Glut. On these bases, we could not provide a clear cause–effect relationship between Cd binding and functional changes in sperm cells. Further studies are required to clarify the possible intracellular accumulation of this specific heavy metal. In addition, we did not consider that a spontaneous sperm acrosome reaction (AR) might have taken place during the analysis, representing a possible bias when considering the membrane binding of Cd. Available data show that early spontaneous AR, taking place within 1–3 h from the ejaculation, as in the case reported here, accounts for 4%–8% of the whole sperm population (Mortimer et al., 1989). Previous studies showed that the exposure Cd had a significant effect on the rate of AR over 24 h of exposure (Marchiani et al., 2019). Taken together, whilst we cannot exclude a proportion of AR spermatozoa, this is unlikely to severely impact our results. Moreover, Cd detection on sperm cells by SEM-EDS was performed adopting a non-random, non-human-chosen, image analysis approach by increasing the likelihood that analysis was more representative of the sample as a whole and that sperm cells had an equal probability of being evaluated, irrespective of their acrosomal status.

In conclusion, our data support a detrimental role of Cd exposure on both motility parameters and fertilization potential of human spermatozoa, likely relying on the stable/irreversible binding of this heavy metal.

## Data Availability

The raw data supporting the conclusion of this article will be made available by the authors, without undue reservation.
