# Supplementary material for: Seminal cadmium affects human sperm motility through stable binding to the cell membrane
Source: Front Cell Dev Biol. 2023 May 18;11:1134304. doi: 10.3389/fcell.2023.1134304 (PMC10232869; doi:10.3389/fcell.2023.1134304)
Supplement: Supplementary file 1 [file Table1.doc]

| **Parameter** | **Not exposed (N=15)**  Mean value ± SD  *[Median, Interquartile range]* | **Exposed (N=30)**  Mean value ± SD  *[Median, Interquartile range]* | **P Value** |
| --- | --- | --- | --- |
| Age (years) | 35.1 ± 6.3  *[34.0, 6.1]*] | 31.2 ± 7.9  *[30.0, 8.3]* | 0.223  *0.251* |
| BMI (kg/m^2^) | 27.3 ± 4.9  *[27.1, 5.0]* | 26.7 ± 4.5  *[27.0, 5.3]*] | 0.684  *[0.711]* |
| Semen volume (mL) | 3.9 ± 1.8  *[3.5, 1.4]* | 4.5 ± 1.7  *[4.45, 2.25]* | 0.279  *0.220* |
| Semen pH | 8.5 ± 0.4  *[8.6, 0.4]* | 8.4 ± 0.3  *[8.4, 0.4]* | 0.621  *0.325* |
| Total Sperm count (10^6^ cells/ejaculate) | 138.4 ± 26.7  *[103.0, 46.8]* | 118.6 ± 105.6  *[87.0, 137.93]* | 0.099  *0.086* |
| Progressive motile sperm fraction (%) | 59.1 ± 9.6  *[56.0, 20.0]* | 52.7 ± 14.5  *[56.0, 24.5]* | 0.130  *0.904* |
| Total motile sperm fraction (%) | 66.3 ± 7.3  *[65.0, 6]* | 59.6 ± 13.6  *[55.0, 18.5]* | **0.037**  ***0.048*** |
| Sperms with normal morphology (%) | 5.8 ± 2.3  *[4.8, 4.0]* | 7.2 ± 3.3  *[7.0, 6.0]* | 0.101  *0.217* |
| Viable sperm cells (%) | 73.0±12.1  *[71.0, 17]* | 71.7±7.6  *[72.1, 9.0]* | 0.765  *0.945* |
| Semen Cd (μg/L) | < LOD  *[n.a.]* | 1.4 ± 0.9  *[7.0, 1.08]* | **0.001**  ***0.001**** |

**Table 1 Demographic and semen parameters of 30 subjects residing in high environmental impact and 15 control subjects residing in low exposure areas**

Abbreviations: standard deviation, SD; body mass index, BMI; Cadmium (Cd). Significant P values are in bold. P value at non-parametric Mann Whitney U test are in *Italic.*
